# Supplementary material for: No Evidence for Associations between men’s Salivary Testosterone and Responses on the Intrasexual Competitiveness Scale
Source: Adapt Human Behav Physiol. 2018 Jun 5;4(3):321–7. doi: 10.1007/s40750-018-0095-2 (PMC6428293; doi:10.1007/s40750-018-0095-2)
Supplement: Supplementary file 1 — (PDF 344 kb) [file 40750_2018_95_MOESM1_ESM.pdf]

Code

No evidence for associations between men’s salivary testosterone and responses on the Intrasexual Competitiveness Scale

Jaimie Torrance, j.torrance.1@research.gla.ac.uk (mailto:j.torrance.1@research.gla.ac.uk)

- Basic Descriptive Information for Sample
  - The number of sessions completed per man
  - Mean age for the sample
- Data Processing
  - Exclude hormone outliers
  - Centre and scale hormones
  - Mean hormone levels
- Intrasexual Competitiveness Analyses
  - Descriptives for Intrasexual Competitiveness Scale
  - Cronbach’s Alpha for Intrasexual Competitiveness Scale
  - Results for LMEM Analysis for Intrasexual Competitiveness Scale
- State Anxiety Analyses
  - Descriptives for State Anxiety Scale
  - Results for LMEM Analysis for State Anxiety Scale

Code

Descriptive statistics and full output for all analyses. This document also includes analyses of reported anxiety levels that are not reported in the main text.

Code

Basic Descriptive Information for Sample

The number of sessions completed per man

Code

sessions <int>	n <int>
1	4
2	2
3	2
4	4
5	47

5 rows

Mean age for the sample

Code

n	mean_age	sd_age	se_age
<dbl>	<dbl>	<dbl>	<dbl>
59	22.06	3.24	0.42

1 row

Code

Data Processing

Exclude hormone outliers

Code

hormone	valid	excluded
<chr>	<int>	<int>
c	264	1
t	261	4

2 rows

Code

Centre and scale hormones

Centre hormones on subject-specific means, and bring values between -0.5 and 0.5 to facilitate calculations in linear mixed effects models. This graph illustrates that testosterone and cortisol values are not skewed.

Code

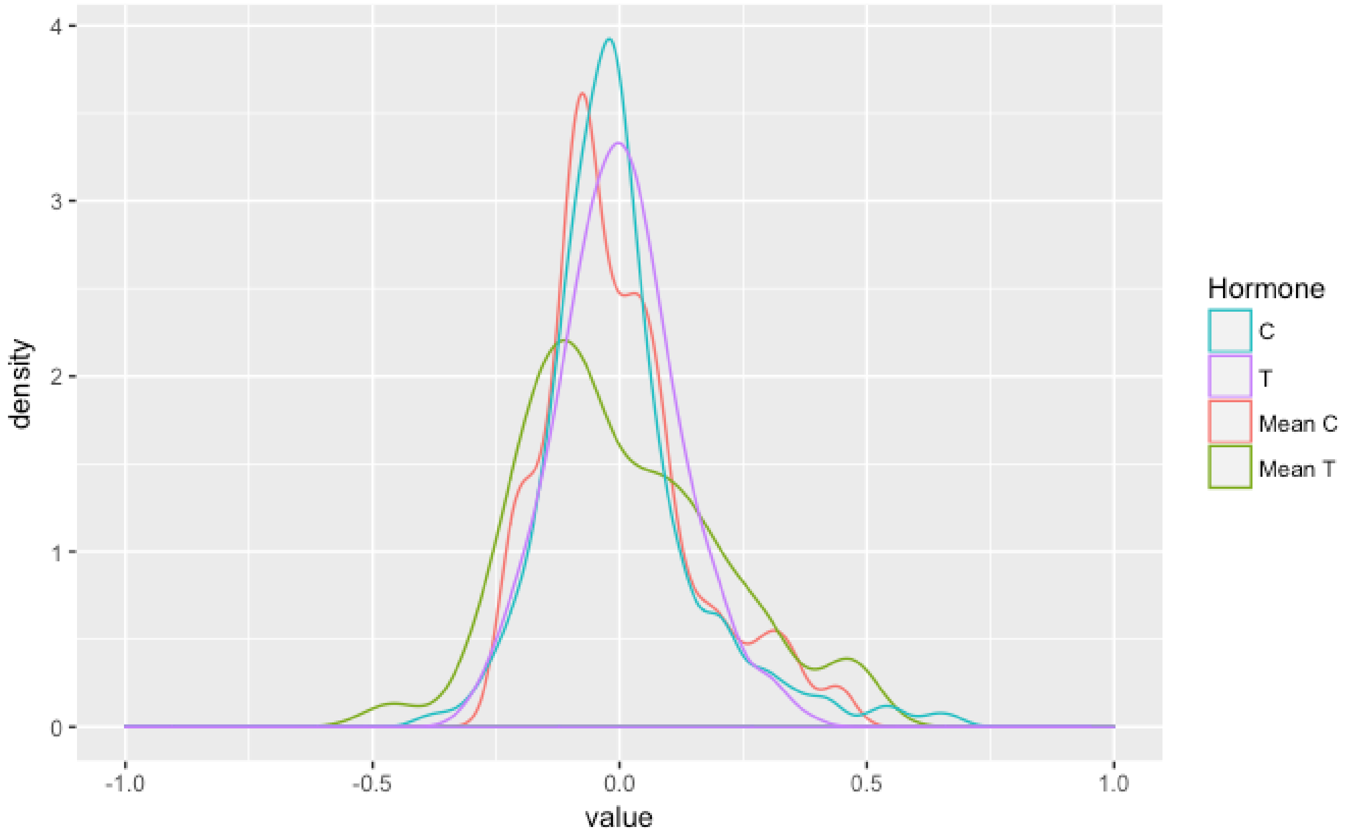

Mean hormone levels

Code

hormone <chr>	mean <dbl>	sd <dbl>	se <dbl>
cort	0.1877	0.1075	0.0066
test	177.5396	42.1505	2.6090

2 rows

Code

Intrasexual Competitiveness Analyses

Descriptives for Intrasexual Competitiveness Scale

Code

mean <dbl>	sd <dbl>	se <dbl>
2.9534	0.9847	0.0607

1 row

Code

Cronbach's Alpha for Intrasexual Competitiveness Scale

Code

```
[1] 0.8634041
```

Results for LMEM Analysis for Intrasexual Competitiveness Scale

Effect <chr>	Estimate <dbl>	Std. Error <dbl>	df <dbl>	t value <dbl>	p <dbl>
(Intercept)	3.035	0.123	59.393	24.634	0.000
test.s	0.053	0.227	38.277	0.235	0.815
cort.s	0.094	0.216	34.618	0.434	0.667
avg_test.s	0.389	0.647	60.287	0.601	0.550
avg_cort.s	-0.221	0.865	61.431	-0.256	0.799
test.s:cort.s	2.565	1.566	162.409	1.637	0.103
avg_test.s:avg_cort.s	-4.421	3.029	59.536	-1.460	0.150

7 rows

State Anxiety Analyses

Descriptives for State Anxiety Scale

Code

mean <dbl>	sd <dbl>	se <dbl>
36.1217	8.8062	0.543

1 row

Code

Results for LMEM Analysis for State Anxiety Scale

Effect <chr>	Estimate <dbl>	Std. Error <dbl>	df <dbl>	t value <dbl>	p <dbl>
(Intercept)	36.556	0.924	55.498	39.569	0.000
test.s	2.967	3.654	45.589	0.812	0.421
cort.s	7.100	2.854	146.327	2.488	0.014

avg_test.s	5.670	4.908	57.793	1.155	0.253
avg_cort.s	-6.165	6.642	60.253	-0.928	0.357
test.s:cort.s	1.094	24.561	18.182	0.045	0.965
avg_test.s:avg_cort.s	-26.526	22.824	56.551	-1.162	0.250

7 rows
